# Assessment of the Quality, Bioactive Compounds, and Antimicrobial Activity of Egyptian, Ethiopian, and Syrian Black Cumin Oils

**DOI:** 10.3390/molecules29214985

**Published:** 2024-10-22

**Authors:** Adel Gabr Abdel-Razek, Minar Mahmoud M. Hassanein, Shimaa Moawad, Amr Farouk, Ahmed Noah Badr, Mohamed Gamal Shehata, Aleksander Siger, Anna Grygier, Magdalena Rudzińska

**Affiliations:** 1 Fats and Oils Department, National Research Center, Dokki, Cairo 12622, Egypt; adelgabr2@gmail.com (A.G.A.-R.); minarmahmoud@gmail.com (M.M.M.H.); 2 Flavor and Aroma Chemistry Department, National Research Center, Cairo 12622, Egypt; shimaa_ahmed199@yahoo.com (S.M.); af.mansour@nrc.sci.eg (A.F.); 3 Food Toxicology and Contaminants Department, National Research Centre, Dokki, Cairo 12622, Egypt; an.badr@nrc.sci.eg; 4 Food Technology Department, Arid Lands Cultivation Research Institute, City of Scientific Research and Technological Applications (SRTA-City), New Borg El-Arab 21934, Egypt; gamalsng@gmail.com; 5 Food Research Section, R&D Division, Abu Dhabi Agriculture and Food Safety Authority (ADAFSA), Abu Dhabi 20602, United Arab Emirates; 6 Faculty of Food Science and Nutrition, Poznań University of Life Sciences, Wojska Polskiego 28, 60-637 Poznań, Poland; aleksander.siger@up.poznan.pl (A.S.); anna.grygier@up.poznan.pl (A.G.)

**Keywords:** *Nigella sativa* oil, phytochemicals, antibacterial activity, antifungal activity, anti-aflatoxigenic impact

## Abstract

Background: The oils obtained from the seeds of *Nigella sativa*, also named black cumin, are rich in bioactive compounds that strengthen immunity and support human health. This study aimed to compare *Nigella sativa* oils pressed from Egyptian (Eg-NSSO), Ethiopian (Et-NSSO), and Syrian (Sy-NSSO) seeds. Methods: The analyzed oils were obtained from a local company. The content of phenolic compounds, tocochromanols, phytosterols, volatile compounds, triglycerides, and fatty acids composition was determined using chromatographic methods. The oxidative stability was determined by Rancimat technique as well as the determination of DPPH and ABTS scavenging activity. As an assessment of bioactivity, the antimicrobial and anti-aflatoxigenic properties of oils were evaluated. Results: Ethiopian oil had highest content of phenolic compounds, flavonoids, phytosterols, and tocochromanols and was characterized by the longest induction period (IP = 7.89 h). The share of thymoquinone was the highest in Ethiopian oil (34.84%), followed by Egyptian (27.36%), then Syrian (22.59%). Ethiopian oil recorded a high antibacterial activity, while Egyptian oil showed a unique antifungal activity against toxigenic fungi. Aflatoxins’ secretion into liquid medium containing NSSO was reduced, especially with Egyptian oil.

## 1. Introduction

Black cumin oil is extracted from the seeds of the flowering plant *Nigella sativa*, which is endemic to dry regions. *Nigella sativa* seed oil (NSSO) has been used for centuries for its medicinal properties. The remarkable properties of NSSO, such as its antioxidant, anti-inflammatory, and antimicrobial effects as well as its potential benefits for digestive, skin, and hair health, have been widely reported [1]. Thymoquinone, thymohydroquinone, and thymol are among the main bioactive chemicals found in NSSO in addition to alkaloids, saponins, vitamins, and others [2]. The NSSO quality and composition could differ based on location, extraction method, and processing procedures [3]. NSSO may be available in various forms, such as cold-pressed, organic, or produced using specialized processes, resulting in differences in flavor, scent, or nutritional content [4]. Different bioactivities and antioxidant potentials were recorded for NSSO based on the origins of cultivation due to differences in fatty acids or bioactive compositions [5].

The traditional NSSO handled in the Mediterranean Basin varies among Egyptian, Ethiopian, and Syrian oils. Egyptian NSSO is regarded for its quality and aromatic, antioxidant, and antibacterial activities, which show valuable efficiency in food storage [6]. The Ethiopian oils exhibit high DPPH radical scavenging and nitric oxide (NO) inhibition activities, which may be related to high levels of thymoquinone and thymol. Syrian samples exhibit high α-glucosidase inhibitory activity, which is correlated with high fatty acid content [5]. Several compounds present in NSSO have demonstrated antimicrobial properties against diverse bacterial strains [1]. These components imply prospective uses in bolstering the immune system and mitigating infections [7]. Among the significant food risks are toxigenic fungi, along with their secondary mycotoxin metabolites. These factors of toxin-producing fungi and pathogens threaten food safety and security [8]. However, implementing natural bioactive substances effectively reduced these hazard sources [9]. Applying these substances raw or utilizing modern application technology could enhance the safety and acceptability of food materials, mainly to regulate for the acceptable limits.

To the best of our knowledge, no studies could be found in the literature dealing with the quantitative differences in the bioactive constituents among NSSO from different origins, especially from Egypt, Ethiopia, and Syria. The main focus was to discover the differences in amounts of volatile components, fatty acids, triacylglycerols, tocopherols, and phytosterols in cold-pressed NSSO of Egyptian, Ethiopian, and Syrian origin. Antimicrobial potency was one of the main differentiation criteria for classifying the NSSO oils. Antioxidant activity and total phenolic compounds illustrate the different oils’ in vitro activity against toxigenic fungi and their mycotoxin production. The functionality of the bioactive constituents of oils also makes sense of the results.

## 2. Results and Discussion

### 2.1. Total Phenolic, Flavonoids, and Oxidative Potency

**Figure 1:**
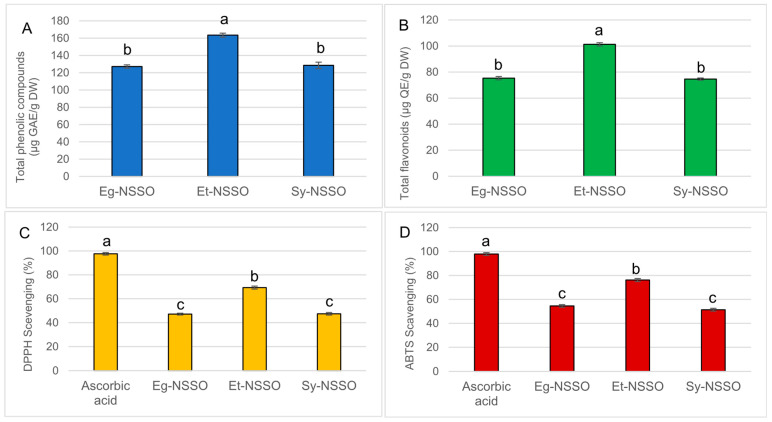
Total phenolic compounds (**A**), flavonoids (**B**), and antioxidant activities determined as DPPH (**C**), ABTS^+^ (**D**) against standard of ascorbic acid. Results are expressed as mean ± SD, means with different letters differ significantly (*n* = 5; *p* ≤ 0.05). Eg-NSSO: Egyptian *Nigella sativa* seed oil; Et-NSSO: Ethiopian *Nigella sativa* seed oil; Sy-NSSO: Syrian *Nigella sativa* seed oil.

The content of phytochemicals in analyzed NSSOs is shown in Figure 1A,B. The total phenolic content in black cumin oils ranged from 127.17 ± 1.52 in Eg-NSSO to 163.50 ± 2.29 μg GAE/g in Et-NSSO. The flavonoid content ranged from 74.63 ± 0.79 in Sy-NSSO to 101.27 ± 1.26 μg QE/g in Et-NSSO.

The antioxidant activity of analyzed oils is presented in Figure 1C,D. The antioxidant activity of the oils was concentration-dependent. The Et-NSSO exhibited a higher antioxidant activity by two methods (DPPH and ABTS) compared to the other two types (Eg-NSSO and Sy-NSSO). Concerning the DPPH, the recorded inhibition percentages of Eg-NSSO and Sy-NSSO close to each other, which may vary in the content of the minor constituents.

Regarding the ABTS inhibition percentages of the examined oils, the results showed that Et-NSSO was the pioneer oil, followed by Eg-NSSO, and Sy-NSSO came last. The results from the two assays emphasized the high antioxidant activity of Et-NSSO; this efficiency could be linked to the variation recorded regarding the total content of phenolics and flavonoids. The antioxidant potency of NSSOs is also thought to be joined with other contents, such as thymoquinone, thymohydroquinone, and thymol, which are known to possess antioxidant properties [10].

The oxidative stability was also measured by Rancimat method, and induction period (IP) was measured at 120 °C. It was found that Et-NSSO was the most stable oil, and its IP was 7.89 h, followed by Eg-NSSO with IP 2.5 h, and then Sy-NSSO, whose IP was 1.98 h. The variability of induction period values may be due to the variation in the contents of volatiles, polyphenols, and tocochromanols [11].

### 2.2. Volatile Compunds

The SPME-GC-MS analysis of the volatile compounds extracted from Egyptian, Ethiopian, and Syrian oils (Table 1) revealed that p-cymene, thymoquinone, and α-thujene were the predominates but were with marked quantitative differences. These variations could result from genetic, seasonal, or seed cultivar effects on the volatiles concentration. The percentage of thymoquinone is one of the essential characteristics of the *N. sativa* volatile fraction due to its well-known biological activities. In the current study, the thymoquinone percentage of the volatile Ethiopian oil was the highest (34.8%), followed by the Egyptian one (27.4%), while the Syrian oil had the lowest thymoquinone share (22.6%). Syrian volatiles were characterized by the highest content of p-cymene (38.8%), α-thujene (17.7%), and carvacrol (3.6%) compared with the Egyptian and Ethiopian volatile fractions (Table 1). The high monoterpenes fraction of the Syrian sample is consistent with the relatively low content of thymoquinone.

The volatiles of analyzed NSSOs varied in thymoquinone and terpene contents, which affect the oil activity and specific potency. The previous findings reported by Edris et al. [12] referred to a volatile constituent composition of the Eg-NSSO that is close to the present evaluation. To our knowledge, a single study by El-Turkmani et al. [13] was found concerning the volatile constituents of *N. sativa* from different locations in Syria, which is consistent with our results; however, nothing has been published about the volatile fraction of Ethiopian oil.

### 2.3. Fatty Acids Composition

The fatty acid composition of three investigated oils is presented in Table 2. The obtained results showed that the major components of fatty acids were linoleic and oleic acids. The linoleic acid dominated in all analyzed oils, and their percentage was 61–62%. The share of oleic acid (n-9) ranged from 21 to 22%. Our samples also contained saturated fatty acids, especially palmitic acid. The percentage of this fatty acid in analyzed NSSO oils was 12%. The share of linolenic acid was very low at 0.2% in all samples. Our results agree with Matthaus and Özcan [14], who studied 14 samples of NSSO from Germany and Turkey. They found that all NSSO samples had higher linoleic acid percentage, followed by oleic acid and palmitic acid, the major saturated fatty acid. In the analyzed oils, eicosanoic and eicosadienoic fatty acids were detected, and their share was 0.2–0.3% and 2.1–2.3%, respectively.

Our results agree with Hassanein et al. [15], who found that the primary fatty acid in Egyptian *Nigella sativa* oil was linoleic rather than oleic acids. At the same time, palmitic acid was the primary saturated fatty acid. These results also agree with Rohman et al. [16], who reported that oleic and linoleic acids are the primary fatty acids comprising NSSO. Farhan et al. [17] studied Saudi oil’s characteristics and fatty acid composition, where the oleic, linoleic, and palmitic acids were the primary fatty acids of *Nigella sativa* oils. Our results are in agreement with Al-Naqeeb et al. [18], where the main fatty acids in NSSO from three regions of Yemen were linoleic, oleic, and palmitic oils. They also showed that extraction method did not affect the fatty acid composition in *Nigella sativa* oils.

### 2.4. Triacylglycerols

The HPLC-ELSD analysis of the triacylglycerol (TAG) molecular species of three *Nigella sativa* oils (Egyptian, Syrian, and Ethiopian) showed the presence of five TAGs containing palmitic, oleic, and linoleic acyl groups designated as P, O, and L, respectively (Table 3). TAGs with lower ECN eluted ahead of those with higher ECN, and the elution sequence within each TAG category with the same ECN (critical pairs) started with the highest number of double bonds and terminated with the lowest unsaturation. The designations of TAGs do not imply the positional acyl distribution in the TAG’s molecules but a mixture of TAG isomers. In each Egyptian, Syrian, and Ethiopian SSO sample, the significant TAGs contained three or two linalyl acyls: LLL, OLL, and PLL, constituting about 60% of the total TAGs. Meanwhile, POL and OLL were found in appreciable amounts.

Farhan et al. [17] showed that Saudi *Nigella sativa* oil was classified as a highly unsaturated oil with 85.7% unsaturated TAGs. The dominant TAGs of Saudi oils were 1-oleoyl-2,3-dilinoleoylglycerol, OLL (37.7%); 1,2,3-trilinoleylglycerol, LLL (35.9%); 1-palmitoyl-2,3-dilinoleylglycerol, PLL (6.7%); 1,2,3-trioleylglycerol, OOO (6.4%); and 1,2-dioleyl-3-linoleylglycerol, OOL (5.7%).

### 2.5. Tocochromanols

The Et-NSSO contains higher amount of total tocochromanols, followed by the Egyptian and Syrian varieties, amounting, respectively, to 40.35, 26.13, and 19.48 mg/100 g (Table 4). There were no significant differences between Eg- and Sy-NSSO in the content of α-tocopherol (α-T), while it was significantly higher in Et-NSSO. According to FDA regulations, we did not have to include other forms of tocochromanols in the vitamin E group, but other forms of tocochromanols can be listed in the ingredient statement for foods [19]. The high content of vitamin E in Et-NSSO is very important for consumers and ought to be labeled on this oil. Just as α-T is a valuable component of oil from the consumer’s point of view, high levels of γ-tocopherol (γ-T) have strong antioxidant properties in oil. Again, Ethiopian oil stands out significantly among the oils tested.

In all analyzed NSSO oils, the content of total tocotrienols was higher than the total tocopherols. The total content of tocotrienols was highest in Et-NSSO, followed by Eg-NSSO and Sy-NSSO. Et-NSSO contained the highest amounts of α-tocotrienol (α-T3), which amounted to 14.33 mg/100 g of the oil, but all *Nigella sativa* oils were characterized by a high level of β-tocotrienon (β-T3). Their content ranged from 14.6 in Et-NSSO to 17.4 mg/100 g in Eg-NSSO (Table 4). γ-Tocotrienol (γ-T3) was not detected in the analyzed oils, contrary to the Saudi NSSO that was shown to contain γ-T3 [17].

Tocotrienols are considered superior to tocopherols for many reasons associated with biological functions related to health and disease. α-, γ-, and δ-tocotrienols were identified to be more effective than α-tocopherol, which was more commonly tested. More recent research studies have revealed that tocotrienols surpass tocopherols by having better antioxidant, antihypertensive, anti-cancer metastatic, anti-inflammatory, anti-hypercholesterolemic, anti-atherogenic, anti-tumor, anti-proliferative, apoptotic, anti-angiogenic, and neuroprotective characteristics documented in a large number of preclinical and clinical studies [20].

### 2.6. Sterols

Phytosterols are considered a significant unsaponifiable fraction in many vegetable oils. They represent an important bioactive compound of plant oils, which is known due to its cholesterol-lowering properties and antioxidation activity. The analysis of phytosterols yielded precise information about the quality and the identity of the oil investigated detected oils and mixtures not identifiable by their fatty acids profile.

The total content of sterols in analyzed oils ranged from 1.84 mg/g in Eg-NSSO to 2.88 mg/g in Et-NSSO (Table 5). This is higher than results obtained by Qian et al. [21], where the total content of sterols in black cumin oil was 0.44 mg/g. In the analyzed *Nigella sativa* seed oils, nine sterols were identified. Cholesterol, the content of which ranged from 0.07 to 0.10 mg/g, probably is formed during the early stages of plant sterols formation in the seeds. In plants, cholesterogenesis has been hypothesized to occur via a similar biosynthetic route and via the same enzymes producing phytosterols or else via a distinct but unknown pathway [22]. In Et-NSSO, the level of cycloartenol was high and amounted to 0.77 mg/g, whereas the content of β-sitosterol was 0.75 mg/g. It is not typical for plant oils and β-sitosterol to dominate in vegetable oils. But cycloartenol, generated by cycloartenol synthase, was described as a precursor of plant sterols [22]. It was also found that Et-NSSO showed the highest content of cholesterol, stigmasterol, β-sitosterol, Δ5 avenasterol, and cycloartenol compared to the other analyzed oils. Sy-NSSO had higher amounts of 24-methylene-cycloartenol, followed by Eg-NSSO and Et-NSSO. It was noticed that there were no significant differences in the content of campesterol in Et-NSSO and Sy-NSSO, which amounted to 0.16 mg/g.

### 2.7. Anti-Pathogenicity and Antifungal Effects of NSSO Types

**Figure 2:**
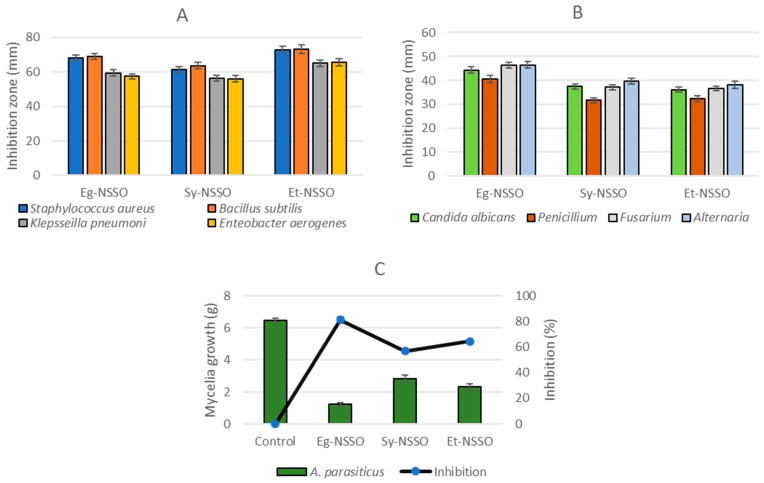
Anti-pathogenicity (**A**), antifungal (**B**), and dissimilarity of aflatoxin-fungi inhibition efficiency (**C**) by *Nigella sativa* seed oils.

The anti-pathogenic effects of *Nigella sativa* oils against the applied strains of Gram-positive or Gram-negative bacteria is presented in Figure 2A. It was noticed that the activity of oils against Gram-positive was greater than against Gram-negative bacteria. The Et-NSSO was shown as the most effective oil against bacteria, followed by the Eg-NSSO, and Sy-NSSO. Again, *B. subtilis* was established as the most sensitive strain to NSSO oil treatments. The antifungal impact of the NSSO oils was evaluated against four fungal strains, and the result reflected a significant inhibition effect. The most effective oil was the Eg-NSSO, particularly against *Fusarium* and *Alternaria* fungi (Figure 2B). Among the bacteria tested, *Penicillium* was the least sensitive strain to inhibition by the NSSO oils. The variation of the inhibition effect between Et-NSSO and Sy-NSSO was not great, and the results against the used four strains seemed to be close.

### 2.8. Aflatoxin Reduction

The reduction of aflatoxin produced by *A. parasiticus* was estimated in a simulated liquid medium (Figure 2C). Significant reduction ratios showed the results for the AFB1, AFB2, AFG1, and AFG2 toxins (Table 6). Firstly, the reduction of *A. parasiticus* mycelia growth was estimated, where the result represented the efficacy of Eg-NSSO in suppressing the fungal mycelia (Figure 2C). The reduction ratio of *A. parasiticus* mycelia ascended in the following order: Sy-NSSO, followed by Et-NSSO, and the Eg-NSSO was the pioneer oil for minimizing aflatoxins’ secretion in the liquid medium.

Aflatoxin reduction of the fungal growth in liquid medium was recorded for Et-NSSO, at 62.83%, 64.03%, 59.92%, and 61.32% for AFB1, AFB2, AFG1, and AFG2, respectively. The values recorded a lower efficiency for Sy-NSSO: 44.45%, 44.76%, 44.57%, and 43.47% for AFB1, AFB2, AFG1, and AFG2, respectively. The highest aflatoxins-diminishing ratios for AFB1, AFB2, AFG1, and AFG2, using the Eg-NSSO as a fungal growth media treatment, were recorded as 64.59%, 64.94%, 62.99%, and 61.87%, respectively.

The data represent a significant reduction regarding the four types of aflatoxin secreted in the growth media of fungi due to the oil’s presence. For AFB1 and AFG2, no significances were observed between the effect of Eg-NSSO and the Et-NSSO. However, a significance was detected in the pioneer impact of Et-NSSO for AFB2 and AFG1 reduction in the medium. The variation recorded for the reduction impact of different oils may be linked to their content of bioactive molecules that could change the metabolic pathways inside the fungal cell system.

## 3. Materials and Methods

### 3.1. Materials

#### 3.1.1. Nigella Sativa Seed Oils (NSSO)

Samples of the Egyptian, Ethiopian, and Syrian NSSO were gifted from a local company specialized in NSSO exportation. Samples were packed in amber bottles with cartoon covers to avoid light and temperature changes. The samples represent the actual state of commercial trading bulk oils, offering an accurate assessment for the investigation. Three bottles of each type of NSSO were taken for analysis, and determinations were made in 3–5 replicates depending on the method of analysis.

#### 3.1.2. Media, Chemicals, and Microorganisms

The ascorbic acid, 5α-cholestane, phytosterols, all solvents, sodium hydroxide, and anhydrous pyridine were purchased from Sigma-Aldrich (St. Louis, MO, USA); while gallic acid, ABTS solution, and DPPH (≥90%) were sourced from Merck KGaA (Darmstadt, Germany). The silylation mixture of BSTFA [*N*,*O*-Bis(trimethylsilyl) trifluoroacetamide] with 1% TMCS (trimethylchlorosilane) was obtained from Fluka Chemie GmbH (Buchs, Switzerland). Standards of alkanes C6–C26 and TAG standards were purchased from Supelco Inc. (Bellefonte, PA, USA).

Applied microbiological media were purchased from HiMedia Laboratories GmbH, Einheusen, Germany. Applied solvents, chemicals, and buffers are HPLC-grade and purchased from Sigma-Aldrich, Darmstadt, Germany. Two bacterial groups were used for the antibacterial evaluations: Gram-positive (G+) and Gram-negative (G−) strains. The G+ strains contain *Staphylococcus aureus* ATCC 6538P and *Bacillus subtilis* ATCC 14579, while *Enterobacter aerogenes* NRRL B199 and *Klebsiella pneumonia* ATCC 13883 are of the G− strains. Bacterial strains were provided by the Laboratory of Microbiology, Agriculture research institute, and National Research Centre. Fungal strains of *Candida albicans* ATCC 14053, *Penicillium chrysogenum* ATCC 48271, *Alternaria alternate* ATCC 11680, and *Fusarium oxysporum* ITEM 1259 were obtained from The ITEM—Agro-Food Microbial Culture Collection, ISPA, CNR, Bari, Italy.

### 3.2. Methods

#### 3.2.1. Total Phenolic, Flavonoids, and Oxidative Activities

To evaluate the total phenolic contents, the Folin–Ciocalteu technique was used as described by Hassanien et al. [23]. The total phenolic contents were estimated using a gallic acid calibration curve and represented as mg of gallic acid equivalent (GAE) per gram of sample (mg GAE/g). The supernatant’s absorbance (510 nm) was measured using a Shimadzu spectrophotometer UV/VIS-1800 (Kyoto, Japan) and was compared to the blank, computed using a catechin calibration curve, and expressed as milligrams of catechin equivalent per gram of sample (mg CE/g).

The antioxidant activity of the extracted materials was evaluated using two assays: DPPH* and ABTS scavenging [24]. For the first analysis, the stable 2,2-diphenyl-1-picrylhydrazyl (DPPH*) was used to measure the extracts’ ability to scavenge free radicals. The total volume of the reaction was 3.0 mL, and the final DPPH* concentration was 200 M. After a 60 min incubation time in the dark, the absorbance at 517 nm was measured using a Shimadzu spectrophotometer UV/VIS-1800 (Kyoto, Japan) and was compared to a pure methanol blank. The percent inhibition of the DPPH free radical was calculated using the following equation:

(1) % inhibition=(Ac−As)/Ac×100

where Ac represents the control reaction absorbance (NSSO-free), and As represents the absorbance with the test compound.

The second test to evaluate antioxidant activity was the 2,2′-Azino-bis (3-ethylbenzothiazoline-6-sulfonic acid) di-ammonium salt radical cation (ABTS^+^). A Shimadzu spectrophotometer UV/VIS-1800 (Kyoto, Japan) measured the absorbance at 700 nm to determine reducing power. Ascorbic acid was used as a positive control, whereas deionized water was used as a blank. A Metrohm Rancimat apparatus model 892 (Metrohm, 4800 Zofingen, Switzerland) was used to determine the induction period of shea butter samples at 120 °C ± 0.1 °C and an air flow of 20 L/h. All analyses were performed in five replicates.

#### 3.2.2. Volatile Compounds

Solid-phase micro-extraction (SPME) was used to extract volatile compounds from the analyzed *Nigella sativa* seed oils. The three-phase fiber (DVB.CAR/PDMS) was used for the absorption of volatiles [25]. The fiber was conditioned in the GC injection port at 270 °C for 4 h and then was exposed to the headspace of samples for 5 min at room temperature. For determination of volatiles, the gas chromatograph Agilent (Santa Clara, CA, USA) 8890 GC System was employed using an HP-5MS fused silica capillary column (30 m, 0.25 mm i.d., and 0.25 mm film thickness) and a mass spectrometer (Agilent 5977B GC/MSD). The oven temperature was originally 50 °C, then regulated from 50 to 200 °C at 5 °C/min and from 200 °C to 280 °C at 10 °C/min, and then held (7 min/280 °C). Helium carried 1.0 mL/min, and 1 μL was injected at split 1:50. The temperature of injection was 230 °C. Electron impact mode (EI) mass spectra were acquired at 70 eV and scanned m/z from 39 to 500 amu. We matched isolated peaks with the NIST mass spectra database, standards, and published data to identify them. The discovered ingredient percentages were calculated from GC peak regions. The retention durations of a homologous sequence of C6–C26 n-alkanes were used to calculate the Kovats index for each molecule and compared to published values. All analyses were performed in three replicates.

#### 3.2.3. Fatty Acids Composition

The fatty acids profile was analyzed using gas chromatography following the AOCS Official Method Ce 1k-07 [26]. Gas chromatograph Trace 1300 (Thermo Scientific, Waltham, MA, USA) with flame ionization detector (FID) was used for analysis. The used column was an SPTM-2560 capillary column (100 m × 0.25 mm × 0.2 µm; Supelco, Bellefonte, PA, USA). The carrier gas was hydrogen (1.5 mL/min). For each analysis, 1 µL of the sample was injected. The oven temperature started from 160 °C (1 min), then increased 6 °C/min to 220 °C, and it remained at this temperature for 17 min. The inlet and detector temperatures were 240 °C. All analyses were performed in three replicates.

#### 3.2.4. Triacylglycerols (TAG)

The HPLC with an ELSD detector (1260 Infinity II, Agilent Technologies, USA) was used for TAG analysis. The column temperature (Infinity Lab Poroshell 120 EC-C18 4.6 mm × 100 mm, 2.7 µm, Agilent Technologies, USA) was 30 °C. The initial composition of the mobile phase was as follows: phase A acetonitrile 80%, phase B dichloromethane 20%; up to 30 min phase change to 55% A and 45% B; then up to 40 min changing the composition of the phase to the initial one and holding it for 10 min. Samples were diluted in dichloromethane. ELSD parameters were as follows: evaporator and nebulizer temperatures—30 °C, the gas flow rate—1.60 SLM, the gain (PM)—1.0. TAGs were identified by comparison of retention time with standards. All analyses were performed in five replicates.

#### 3.2.5. Tocochromanols

The tocochromanols content was determined following AOCS Ce 8-89 (1995) [27], using an HPLC system (Shimadzu Corporation, Kyoto, Japan) consisting of a pump (LC-10ADvp), a degasser (DGU-14A), a low-pressure gradient unit (FCV-10ALvp), a system controller (SCL-10Avp), an auto injector (SIL-10AF), a column oven (CTO-10ASvp), and a fluorescence detector (RF-10AXL) in reversed-phase mode, which was also equipped with a Li-Chrosorb Si60 column (250 mm × 4.6 mm; 5 µm; Merck KGaA, Darmstadt, Germany). All analyses were performed in five replicates.

#### 3.2.6. Phytosterols

The content of phytosterols was analyzed according to AOCS Official Method Ch 6-91 [28]. Analyzed samples were saponified with 1 M methanolic KOH for 18 h. Then, phytosterols were extracted with hexane/methyl *tert*-butyl ether (1:1, *v*/*v*). The extracts were evaporated, and samples were silylated using STFA + 1% TMCS. Gas chromatograph HP 6890 series II Plus (Hewlett Packard, Palo Alto, CA, USA) with flame-ionization detector was used for separation. The apparatus was equipped with DB-35MS capillary column (25 m × 0.20 mm, 0.33 μm; J&W Scientific, Folsom, CA, USA). A sample of 1.0 μL was injected in splitless mode. The initial oven temperature was 100 °C for 5 min, then the temperature was programmed to 250 °C at 25 °C/min, held for 1 min, then further programmed to 290 °C at 3 °C/min, and held for 20 min. The carrier gas was hydrogen (1.5 mL/min). Phytosterols were identified by comparison of retention data with standards and using GC/MS technique on 7890A/5975C VL MSD with Triple-Axis Detector (Agilent Technologies Inc., Santa Clara, CA, USA) using the same chromatographic conditions as described for GC-FID. All analyses were performed in five replicates.

#### 3.2.7. Oxidative Stability

For determination of oxidative stability, a Rancimat 743 Metrohm apparatus (Herisau, Switzerland) was used. An oil sample of 2.5 ± 0.01 g was oxidized with a heating temperature of 120 °C and an air flow of 20 L h^−1^. The volatile products that formed from the oxidation reaction were soluble in 0.06 L of distilled water. The induction time was measured in hours and was recorded automatically by 743 Rancimat software 1.1 with an accuracy of 0.01 [29]. All analyses were performed in five replicates.

#### 3.2.8. Antimicrobial Activity

a Antibacterial activity

The well-diffusion assay was applied to assess the antimicrobial efficiency of investigated NSSOs. The activities were evaluated versus bacterial pathogens of Gram-negative and Gram-positive strains. One milliliter of bacterial strains suspension was inoculated in the nutrient agar medium (4.21 × 10^6^ CFU/mL). After media consolidating, a cork-borer tool was utilized to fill the wells with 100 µL of (NSSO for treatment, DMSO for negative control, and standard antibiotic for positive control) for the assay performance of well-diffusion assay. The inhibition effect was represented as a millimeter diameter for the zone inhibition performed by applying NSSO on the agar media.

b Antifungal activity

Four strains of the toxigenic fungi were utilized to investigate the efficacy of the three NSSO types as fungal growth inhibitors. The suppression effect of the NSSO was determined using the well-diffusion assay and the simulated liquid media of fungal growth. The fungal spores were prepared in a tween-water solution, where the spore count was adjusted before being inoculated using a hemocytometer slide counter. The NSSOs were tested at a concentration of 100 µg and loaded into the examined wells for efficacy evaluations. The results of the examined NSSO against the control (control-positive for Nystatin antifungal; control-negative for sterile saline) were expressed as millimeters of zone inhibition (mm), whereas a greater zone inhibition area showed a more sensitive strain against the applied NSSO type. The minimal inhibitory and fungicidal concentrations were assessed according to the methodology described previously [30].

#### 3.2.9. Activity to Reduce Aflatoxins

A spore suspension was prepared for *A. parasiticus* ITEM 11 that was adjusted at 1.21 × 10^3^ CFU/mL for the inoculation step. Five groups of media-growth flasks containing YES media were designed as follows:

G1: Control treatment contains fungal spores.

G2: Media contained fungal spores and 100 µg of the Eg-NSSO.

G3: Media contained fungal spores and 100 µg of Et-NSSO.

G4: Media contained fungal spores and 100 µg of Sy-NSSO.

G5: Media had fungal spores and 100 µg of Nystatin as a standard antifungal.

The flasks were of 500 mL capacity and contained 200 mL YES media as the standard liquid media utilized to support toxigenic fungal growth. Flasks were incubated in parallel in two sections (Section 1: to evaluate fungal growth activity; Section 2: to evaluate toxin production reduction). The incubation conditions were adjusted for each section (Section 1 at 22 ± 1 °C/RH: 85%/5 days; Section 2 at 28 ± 2 °C/RH: 70%/10 days). By the end of the incubation time, the mycelia dry weight for each treatment was recorded, and the growth inhibition was calculated against the fungal growth of the control. In contrast, aflatoxin reduction was determined in the liquid media of Sec2 flasks after extraction using quantitative analyses of a pre-calibrated fluorometer (VICAM Se-ries™, 4EX Fluorometer, Watertown, MA, USA; the LOD 1.0 ng/L) [9]. All analyses were performed in five replicates.

#### 3.2.10. Statistical Analysis

The experiments were carried out in triplicate, all analyses were performed in three or five replicates, and the results are herein expressed in means ± standard deviations (SD). The software of the SPSS program V.16 was applied for the analyses of the results using the analysis of variances (ANOVA), and Duncan’s multiple-range test (*p* < 0.05) was used to evaluate whether the mean values differed significantly.

## 4. Conclusions

Qualitative and quantitative studies as well as antibacterial and antifungal properties and reduced aflatoxin production showed the variation in *Nigella sativa* oils. The analyzed oil from Ethiopia had the highest content of phenolic compounds, flavonoids, thymoquinone, phytosterols including sitosterol and cycloartenol and tocotrienols, as well as total tocochromanols. The results showed a significant effect of oilseed quality on the properties of the finished oil product. In addition, it was shown that the analyzed oil from Ethiopia had the highest oxidative stability. The composition of fatty acids and triacylglycerols was similar in all the oils tested and was characterized by a high proportion of linoleic, oleic, and palmitic acids.

The major phytochemicals in NSSOs, namely β-tocopherol, β-tocotrienol, β-sitosterol, and cycloartenol, showed drug-likeness properties. These findings were emphasized by the in vitro results obtained in the simulated media with aflatoxin production, where oil doses showed a reduction ratio for the four aflatoxin fractions (AFB1, AFB2, AFG1, and AFG2). These findings open perspectives for using NSSO bioactive components as nutritional and functional compounds with antibacterial and anti-aflatoxigenic properties.

## Figures and Tables

**Table 1:** Identification the volatile compounds of *N. sativa* seed oils using GC-MS.

S/N	Compound	RI ^a^	LRI ^b^	Area (%)			Identification Method ^c^
				Eg-NSSO	Sy-NSSO	Et-NSSO	
1	α-Thujene	926	930	15.9 ± 1.9 ^a^	17.7 ± 1.2 ^a^	13.1 ± 1.0 ^b^	RI, MS, STD
2	α-Pinene	935	939	4.8 ± 0.5 ^a^	ND	3.9 ± 0.3 ^b^	RI, MS, STD
3	Sabinene	969	975	2.4 ± 0.3 ^a^	ND	1.9 ± 0.2 ^a^	RI, MS, STD
4	β-Pinene	973	979	4.3 ± 0.4 ^a^	ND	3.7 ± 0.3 ^a^	RI, MS, STD
5	α-Terpinene	1013	1017	0.8 ± 0.2 ^a^	ND	0.3 ± 0.0 ^b^	RI, MS
6	p-Cymene	1019	1024	28.2 ± 2.3 ^b^	38.8 ± 2.2 ^a^	26.7 ± 2.1 ^b^	RI, MS, STD
7	D-Limonene	1033	1029	3.4 ± 0.4 ^a^	ND	2.6 ± 0.2 ^a^	RI, MS, STD
8	γ-Terpinene	1055	1059	2.2 ± 0.3 ^a^	ND	ND	RI, MS
9	*cis*-4-Methoxy thujane	1080	1088	0.9 ± 0.2 ^b^	2.7 ± 0.3 ^a^	1.1 ± 0.1 ^b^	RI, MS
10	*trans*-4-Methoxy thujane	1105	1110	4.9 ± 0.5 ^b^	1.9 ± 0.2 ^c^	5.9 ± 0.5 ^a^	RI, MS
11	iso-3-Thujanol	1129	1138	0.5 ± 0.1 ^b^	0.8 ± 0.1 ^a^	0.4 ± 0.1 ^b^	RI, MS
12	Terpinen-4-ol	1169	1177	0.4 ± 0.1 ^c^	1.3 ± 0.1 ^a^	0.7 ± 0.0 ^b^	RI, MS
13	α-Terpineol	1189	1188	1.0 ± 0.2 ^a^	0.8 ± 0.1 ^b^	1.1 ± 0.1 ^a^	RI, MS
14	Carvone	1240	1243	0.4 ± 0.1 ^a^	ND	ND	RI, MS
15	Thymoquinone	1255	1252	27.4 ± 2.0 ^b^	22.6 ± 1.8 ^c^	34.8 ± 2.9 ^a^	RI, MS, STD
16	Carvacrol	1292	1299	1.3 ± 0.2 ^b^	3.6 ± 0.3 ^a^	1.7 ± 0.1 ^b^	RI, MS, STD
17	α-Longipinene	1350	1352	ND	ND	0.4 ± 0.0 ^a^	RI, MS
18	Longifolene	1409	1407	1.1 ± 0.2 ^b^	0.9 ± 0.1 ^b^	1.7 ± 0.1 ^a^	RI, MS

^a^—RI, retention indices calculated on DB-5 column using alkane’s standards; ^b^—LRI, retention indices according to literature; ^c^—confirmed by comparison with the retention indices, the mass spectrum of the authentic compounds, and the NIST mass spectra library data; ND—not detected; Eg-NSSO: Egyptian *Nigella sativa* seed oil; Et-NSSO: Ethiopian *Nigella sativa* seed oil; Sy-NSSO: Syrian *Nigella sativa* seed oil. Results are expressed as mean ± SD (*n* = 3; *p* ≤ 0.05). The data with the same superscript letter are not significantly different for each row.

**Table 2:** Fatty acid composition of *Nigella sativa* seed oils from different regions.

Fatty Acids	Eg-NSSO	Sy-NSSO	Et-NSSO
Palmitic (16:0)	11.8 ± 0.1 ^a^	11.8 ± 0.1 ^a^	11.6 ± 0.1 ^b^
Stearic (18:0)	2.6 ± 0.1 ^ab^	2.7 ± 0.1 ^a^	2.5 ± 0.1 ^b^
Oleic (18:1 n-9)	21.0 ± 0.1 ^c^	22.1 ± 0.1 ^a^	21.8 ± 0.1 ^b^
Linoleic (18:3 n-6)	62.3 ± 0.2 ^a^	60.6 ± 0.3 ^c^	61.4 ± 0.3 ^b^
Linolenic (18:3 n-3)	0.2 ± 0.0 ^a^	0.2 ± 0.0 ^a^	0.2 ± 0.0 ^a^
Eicosaenoic (20:1)	0.2 ± 0.0 ^b^	0.3 ± 0.0 ^a^	0.3 ± 0.0 ^a^
Eicosadienoic (20:2)	2.1 ± 0.1 ^b^	2.2 ± 0.1 ^a^	2.3 ± 0.1 ^a^

Results are expressed as mean ± SD (*n* = 3; *p* ≤ 0.05). The data with the same superscript letter are not significantly different for each column. Eg-NSSO: Egyptian *Nigella sativa* seed oil; Et-NSSO: Ethiopian *Nigella sativa* seed oil; Sy-NSSO: Syrian *Nigella sativa* seed oil.

**Table 3:** Triacylglycerol profile of *Nigella sativa* oils from Egypt, Ethiopia, and Syria (%).

TAGs	Eg-NSSO	Sy-NSSO	Et-NSSO
POL	12.9 ± 0.4 ^b^	13.7 ± 0.2 ^a^	12.8 ± 0.2 ^b^
PLL	19.7 ± 0.5 ^a^	19.0 ± 0.5 ^a^	18.6 ± 0.1 ^a^
OOL	13.5 ± 0.1 ^b^	15.1 ± 0.2 ^a^	14.4 ± 0.2 ^a^
OLL	19.7 ± 0.2 ^a^	19.1 ± 0.1 ^b^	19.2 ± 0.1 ^b^
LLL	19.7 ± 0.1 ^a^	17.7 ± 0.3 ^c^	18.8 ± 0.2 ^b^

P—palmitic acid; O—oleic acid; L—linoleic acid; the data with the same superscript letter are not significantly different for each row; Eg-NSSO: Egyptian *Nigella sativa* seed oil; Et-NSSO: Ethiopian *Nigella sativa* seed oil; Sy-NSSO: Syrian *Nigella sativa* seed oil.

**Table 4:** The content of tocochromanols in *Nigella sativa* seed oils from Egypt, Syria, and Ethiopia (mg/100 g).

Tocochromanols (mg/100 g)	Eg-NSSO	Sy-NSSO	Et-NSSO
α-Tocopherol	1.03 ± 0.24 ^b^	1.21 ± 0.18 ^b^	1.83 ± 0.37 ^a^
β-Tocopherol	2.31 ± 0.46 ^b^	1.14 ± 0.22 ^c^	5.12 ± 0.84 ^a^
γ-Tocopherol	1.01 ± 0.21 ^b^	0.36 ± 0.16 ^c^	4.14 ± 0.54 ^a^
δ-Tocopherol	0.06 ± 0.02 ^c^	0.13 ± 0.01 ^b^	0.16 ± 0.01 ^a^
α-Tocotrienol	4.09 ± 0.37 ^b^	1.20 ± 0.44 ^c^	14.33 ± 0.28 ^a^
β-Tocotrienol	17.40 ± 1.26 ^a^	15.40 ± 1.02 ^a^	14.64 ± 0.94 ^b^
δ-Tocotrienol	0.25 ± 0.03 ^a^	0.06 ± 0.01 ^c^	0.14 ± 0.08 ^b^
Total	26.13 ± 2.59 ^b^	19.48 ± 2.04 ^c^	40.35 ± 3.06 ^a^

Results are expressed as mean ± SD (*n* = 5; *p* ≤ 0.05). The data with the same superscript letter are not significantly different for each row. Eg-NSSO: Egyptian *Nigella sativa* seed oil; Et-NSSO: Ethiopian *Nigella sativa* seed oil; Sy-NSSO: Syrian *Nigella sativa* seed oil.

**Table 5:** The content of sterols in *Nigella sativa* seed oils from Egypt, Syria, and Ethiopia (mg/g).

Phytosterols (mg/g)	Eg-NSSO	Sy-NSSO	Et-NSSO
Cholesterol	0.08 ± 0.01 ^b^	0.08 ± 0.01 ^b^	0.10 ± 0.01 ^a^
Campesterol	0.13 ± 0.01 ^b^	0.16 ± 0.01 ^a^	0.16 ± 0.01 ^a^
Stigmasterol	0.16 ± 0.01 ^c^	0.21 ± 0.02 ^b^	0.26 ± 0.2 ^a^
β-Sitosterol	0.52 ± 0.02 ^b^	0.75 ± 0.03 ^a^	0.75 ± 0.04 ^a^
Sitostanol	0.07 ± 0.00 ^b^	0.09 ± 0.02 ^b^	0.17 ± 0.03 ^a^
Δ5-Avenasterol	0.11 ± 0.02 ^c^	0.15 ± 0.02 ^b^	0.23 ± 0.02 ^a^
Cycloartenol	0.40 ± 0.04 ^b^	0.45 ± 0.03 ^b^	0.77 ± 0.05 ^a^
Δ7-Avenasterol	0.11 ± 0.02 ^b^	0.17 ± 0.02 ^a^	0.18 ± 0.02 ^a^
24 Methylene-cycloartenol	0.27 ± 0.02 ^b^	0.32 ± 0.01 ^a^	0.26 ± 0.03 ^b^
Total	1.85 ± 0.02 ^c^	2.38 ± 0.08 ^b^	2.88 ± 0.10 ^a^

Results are expressed as mean ± SD (*n* = 5; *p* ≤ 0.05). The data with the same superscript letter are not significantly different for each column. Eg-NSSO: Egyptian *Nigella sativa* seed oil; Et-NSSO: Ethiopian *Nigella sativa* seed oil; Sy-NSSO: Syrian *Nigella sativa* seed oil.

**Table 6:** Aflatoxin reduction in simulated medium of *A. parasiticus* fungi containing individual doses of *Nigella sativa* oils.

	Control	Eg-NSSO	R (%)	Sy-NSSO	R (%)	Et-NSSO	R (%)
AFB_1_	157.21 ± 2.54 ^a^	55.67 ± 1.27 ^c^	64.59	87.33 ± 2.55 ^b^	44.45	58.44 ± 2.39 ^c^	62.83
AFB_2_	160.33 ± 1.67 ^a^	51.26 ± 2.58 ^d^	64.94	88.56 ± 2.27 ^b^	44.76	57.67 ± 1.55 ^c^	64.03
AFG_1_	130.57 ± 2.49 ^a^	48.33 ± 3.11 ^d^	62.99	72.38 ± 2.74 ^b^	44.57	52.33 ± 1.81 ^c^	59.92
AFG_2_	130.22 ± 2.66 ^a^	49.67 ± 2.08 ^c^	61.87	73.61 ± 1.89 ^b^	43.47	50.37 ± 1.28 ^c^	61.32

Results are expressed in mean ± SD (*n* = 3; *p* ≤ 0.05); %R: the reduction percentage of aflatoxin-producing amount extracted from simulated media containing *Nigella sativa* oils compared to the control media (fungi without oil). The data with same superscript letter are not significantly different for each raw: Eg-NSSO: Egyptian *Nigella sativa* seed oil; Et-NSSO: Ethiopian *Nigella sativa* seed oil; Sy-NSSO: Syrian *Nigella sativa* seed oil.

## Data Availability

Dataset available on request from the authors.
